# Fatigue and brain arousal in patients with major depressive disorder

**DOI:** 10.1007/s00406-020-01216-w

**Published:** 2020-12-04

**Authors:** Galina Surova, Christine Ulke, Frank Martin Schmidt, Tilman Hensch, Christian Sander, Ulrich Hegerl

**Affiliations:** 1 Department of Psychiatry and Psychotherapy, University of Leipzig Medical Center, Semmelweisstrasse 10, 04103 Leipzig, Germany; 2 IUBH International University, Erfurt, Germany; 3 Depression Research Center, German Depression Foundation, Leipzig, Germany; 4 Department of Psychiatry, Psychosomatic Medicine and Psychotherapy, Goethe University Frankfurt am Main, Frankfurt am Main, Germany

**Keywords:** Fatigue, Depression, EEG, Brain arousal regulation, Neurophysiology

## Abstract

Fatigue is considered a key symptom of major depressive disorder (MDD), yet the term lacks specificity. It can denote a state of increased sleepiness and lack of drive (i.e., downregulated arousal) as well as a state of high inner tension and inhibition of drive with long sleep onset latencies (i.e., upregulated arousal), the latter typically found in depression. It has been proposed to differentiate fatigue along the dimension of brain arousal. We investigated whether such stratification within a group of MDD patients would reveal a subgroup with distinct clinical features. Using an automatic classification of EEG vigilance stages, an arousal stability score was calculated for 15-min resting EEGs of 102 MDD patients with fatigue. 23.5% of the patients showed signs of hypoarousal with EEG patterns indicating drowsiness or sleep; this hypoaroused subgroup was compared with remaining patients (non-hypoaroused subgroup) concerning self-rated measures of depressive symptoms, sleepiness, and sleep. The hypoaroused subgroup scored higher on the Beck Depression Inventory items “loss of energy” (*Z* = − 2.13, *p* = 0.033; *ɳ*^2^ = 0.044, 90% CI 0.003–0.128) and “concentration difficulty” (*Z* = − 2.40, *p* = 0.017; *ɳ*^2^ = 0.056, 90% CI 0.009–0.139), and reported higher trait and state sleepiness (*p* < 0.05) as compared to the non-hypoaroused group. The non-hypoaroused subgroup, in contrast, reported more frequently the presence of suicidal ideation (Chi^2^ = 3.81, *p* = 0.051; *ɳ*^2^ = 0.037, 90% CI 0.0008–0.126). In this study, we found some evidence that stratifying fatigued MDD patients by arousal may lead to subgroups that are pathophysiologically and clinically more homogeneous. Brain arousal may be a worth while target in clinical research for better understanding the mechanisms underlying suicidal tendencies and to improve treatment response.

**Electronic supplementary material:**

The online version of this article (10.1007/s00406-020-01216-w) contains supplementary material, which is available to authorized users.

## Introduction

The World Health Organization estimates that major depressive disorder (MDD) is the leading cause of the global burden of disease [1]. To date, a major shortcoming of the classification systems for MDD is the lack of methods to identify pathophysiologically and clinically more homogeneous subgroups. Further, there is a lack of semantic clarity regarding its key symptoms [2] which is particularly true when considering fatigue—a core symptom of depression according to ICD-10 and one of the diagnostic criteria in DSM-5. Over 90% of depressed patients complain about fatigue; it is a highly prevalent prodromal and residual symptom of MDD, which has significant effects on functional outcomes (see [3] for review). However, fatigue is also commonly reported in the context of a variety of inflammatory and immunological processes (e.g., [4]).

It has been proposed to differentiate two subtypes of fatigue, namely hyper- and hypoaroused fatigue, based on clinical phenomenology (see Table 1; [5, 6] for detailed explanation). According to this model, fatigue in the context of typical depression is often associated with inner tension and inhibition of drive—a state with an upregulated brain arousal. This is supported by clinical studies demonstrating hyperarousal in depression, as evidenced by hypothalamic–pituitary–adrenal (HPA) axis hyperactivity [7, 8], prolonged sleep onset latencies [9, 10], or increases in heart rate and skin conductance [11]. In contrast, fatigue in the context of inflammatory and immunological disorders or cancer is, according to the model, a state with downregulated arousal. This is corroborated by the finding of an underactive HPA axis in some disorders associated with fatigue (e.g., cancer-related fatigue [12]; for review, see [13]), increased daytime sleepiness (e.g., cancer-related fatigue [14]; multiple sclerosis [15]) and short sleep onset latencies [15]. However, there is first evidence that hyperarousal in these conditions is associated with a higher depression score [16].

**Table 1:** Proposed features distinguishing between two types of fatigue according to the regulation of brain arousal

	Hypoaroused fatigue	Hyperaroused fatigue
Drive	Lack of drive/anergic state [17]	Inhibition of drive/retardation [18]
Daytime wakefulness	Excessive daytime sleepiness [14] short sleep latency in the Multiple Sleep Latency Test [19]	Not sleepy, long sleep latency in the Multiple leep atency Test [9]
Wakefulness regulation	Unstable [43], short sleep latency	Hyperstable [37], prolonged sleep latency
Hypothalamic–pituitary–adrenal axis activity	Decreased, blunted [12,20]	Increased [7, 19, 20]
Sleep disturbances	Predict fatigue severity [21]	Not associated with fatigue severity [21]
Positive treatment response	Psychostimulants [22] dopaminergic/noradrenergic antidepressants [23, 24]	Antidepressants [46]

A modified version of this table was first published in [5]

The suggested distinction assumes differences in the underlying pathophysiology that may have implications for treatment—fatigue syndromes with an unstable arousal regulation might respond to drugs with wakefulness stabilizing properties like psychostimulants, whereas antidepressants might be more effective for conditions with a hyperstable arousal regulation. Moreover, the suggested heterogeneity may at least partly explain the inconsistent effect of the pharmacological treatment on fatigue which has been reported for MDD [3], multiple sclerosis [22] or cancer [25].

The term brain arousal used herein denotes global functional states of the central nervous system (CNS). At the behavioral level, brain arousal levels are associated with different degrees of wakefulness, ranging from alert wakefulness to deep sleep [26]. A successful situational adaptation to changing environmental conditions requires an adequate regulation of arousal; arousal increases in threatening situations and decreases under rest. Besides environmental conditions substantially affecting the level of arousal, stable interindividual differences are known [27, 28], and a genetic basis has been proposed [29, 30]. A growing body of evidence suggests that brain arousal regulation is a fundamental neurophysiological process (for review see [26, 31]). Furthermore, it is one of the five domains to consider for creating meaningful clinical subgroups in psychiatric research proposed by the US-American National Institute of Mental Health (NIMH) in their Research Domain Criteria Project (RDoC; [32]).

The level and the regulation of brain arousal can be assessed using an electroencephalogram (EEG) under resting condition with closed eyes. To objectively assess arousal regulation, an algorithm was developed that allows the reliable classification of EEG vigilance stages [27] within multichannel resting EEG recordings (Vigilance Algorithm Leipzig (VIGALL 2.1); for a detailed description, see [33]). VIGALL has been validated with simultaneous EEG–PET and EEG–fMRI studies (for review, see [34]) and by relating EEG vigilance stages to parameters of the autonomic nervous system [28, 35, 36]. Using this approach, arousal dysregulations have been found in patients with affective disorders and ADHD [10, 37–40]. In patients with MDD an upregulated arousal regulation, compared to healthy controls during a resting state EEG, is a robust finding [37, 41, 42]. In contrast, patients with cancer–related fatigue have been shown to have a downregulation of arousal, with more rapid declines to lower arousal levels than healthy controls during a resting-state EEG [43].

Besides frequently found hyperarousal in MDD, signs of hypoarousal (e.g., excessive daytime sleepiness, hypersomnia) have also been reported (e.g., [44]). In the present study, we set out to examine whether stratifying MDD patients with fatigue by brain arousal would reveal subgroups which differ concerning depressive symptomatology, daytime trait and state sleepiness, and sleep characteristics.

## Materials and methods

### Subjects

Archival records of depressed in- and outpatients admitted to the Psychiatric Department at the University Hospital Leipzig between 08/2012 and 12/2014 were screened. Included were subjects with age ≥ 18, a diagnosis of MDD with a current depressive episode, who had filled out the multidimensional fatigue inventory (MFI-20 [47]; only patients admitted to our department for the first time filled out the MFI-20). From the 141 initially eligible patients, 132 (93.6%) fulfilled the MFI cut-off score criteria (see below for the cut-off definition). Thereof, we excluded patients with comorbid psychiatric (DSM-IV axis I), major somatic or neurological disorders (diagnoses had been confirmed by a senior physician); diseases associated with fatigue (e.g., obesity (BMI ≥ 30 kg/m^2^), HIV, rheumatic disorders, advanced cancer); current z-hypnotics or benzodiazepine treatment; a history of head injury with loss of consciousness over 1 h; alcohol abuse within the past 6 months or use of illegal drugs; pathological EEG and those containing more that 15% artefactual segments; more than 2 missing items per questionnaire. In total, 102 patients were included into the study. Part of the dataset has previously been published [46].

### Questionnaires

To evaluate fatigue, depressive symptoms, sleepiness and sleep characteristics, the following self-report instruments were filled by the patients immediately before the EEG recording:

- Multidimensional Fatigue Inventory (MFI-20; [47]) is a 20-item questionnaire covering five fatigue dimensions: general fatigue, physical fatigue, mental fatigue, reduced activity and reduced motivation. Dimensional scores range between 4 and 20, with higher scores indicating higher degree of fatigue. As the MFI-20 has not yet been standardized specifically for MDD patients, we used norm values obtained from a sample representative of the German population [48]. The influence of age and sex on fatigue scores was taken into account, since the norm values are stated for both sexes and three age groups. Only patients with scores exceeding the 75th percentile on the dimension of general fatigue were included into the study.
- Beck Depression Inventory version II (BDI-II; [49]) is a 21-item questionnaire, assessing the severity of depression; except for items 16 and 18, each item has four possible responses, ranging in intensity from 0 to 3; the sum score ranges from 0 to 63.
- Epworth Sleepiness Scale (ESS; [50]) is an 8-item questionnaire, assessing *trait daytime sleepiness*. On a 4-point scale (0–3), respondents are asked to rate their usual probability of dozing off while engaged in eight different activities (e.g., “sitting and reading”, “as a passenger in a car for an hour without a break”, “sitting quietly after a lunch without alcohol”). The sum score ranges between 0 and 24 with scores over 10 indicating mild excessive daytime sleepiness [51].
- Stanford Sleepiness Scale (SSS; [52]) is a questionnaire assessing *state sleepiness*, with a seven-point rating scale: 1 (feeling active, vital, alert); 2 (functioning at high levels, but not at peak; able to concentrate); 3 (awake, but relaxed; responsive but not fully alert); 4 (somewhat foggy, let down); 5 (foggy; losing interest in remaining awake; slowed down); 6 (sleepy, woozy, fighting sleep; preferring to lie down); 7 (no longer fighting sleep, sleep onset soon).
- Multiple items of German sleep inventory (SF-A/R; [53]) were used to estimate sleep quality and total time in bed the night before the EEG recording. The sleep quality score, ranging from 1 (impaired) to 5 (excellent), consists of items assessing sleep onset latency, quantity and duration of the time awake, early awakening and overall sleep characteristics (e.g., steady, deep, undisturbed). Total time in bed according to SF-A/R is calculated from items assessing time of going to sleep in the evening and time of getting up in the morning.

### EEG recording and preprocessing

The recordings took place between 8 am and 3 pm in a dimly lit booth with sound attenuation; participants were instructed to close the eyes, relax and not fight against sleep pressure. Fifteen minutes of resting eyes-closed EEG was recorded using a 40-channel QuickAmp amplifier (Brain Products GmbH, Gilching, Germany) from 31 monopolar referenced electrodes according to an extended international 10–20 system with impedances kept below 10 kΩ and at a sampling rate of 1 kHz. A total of four bipolar referenced electro-oculogram (EOG) electrodes were placed above and below the right eye and at the canthus of each eye. Data were recorded and preprocessed using the BrainVision Analyzer 2.0 software (Brain Products GmbH, Gilching, Germany). Preprocessing included: filtering (70 Hz low-pass, 0.5 Hz high-pass, 50 Hz notch filter with ± 2 Hz range); rejection of periods with open eyes based on visual EOG screening; rejection of eye movement and muscle artifacts using independent component analysis (ICA). Remaining muscle/sweat/movement artifacts were excluded from the analysis after visual inspection; thereafter, data were re-referenced to a common average.

Brain arousal regulation was assessed using the freely available VIGALL 2.1 algorithm (available at https://research.uni-leipzig.de/vigall/), which attributes one of seven EEG vigilance stages to each 1-s EEG segment. EEG vigilance stages range from active wakefulness to sleep onset (see Table 2 for scoring criteria). Consequently, using scoring criteria presented in Table 3, we calculated an arousal stability score for each subject, indicating the degree of arousal decline. Referring to the replicated finding of a hyperstable arousal regulation in depressed patients, who typically do not reach states of drowsiness and sleep in a 15-min resting EEG, we divided the patients into two groups: patients reaching EEG vigilance stages indicating arousal states of increased drowsiness and sleep (“hypoaroused fatigue”; arousal stability score ≤ 6) and patients not reaching these states (“non-hypoaroused fatigue”; arousal stability score ≥ 7).

**Table 2:** Scoring criteria of EEG vigilance stages (see the VIGALL 2.1 manual [33] for detailed description); higher scores correspond to higher arousal levels

EEG characteristic	VIGALL stage classification	Stage score
Low amplitude, predominantly beta EEG (12–25 Hz) without horizontal SEM	0	7
Alpha frequency (8–12 Hz), dominant in occipital regions	A1	6
Alpha frequency (8–12 Hz), dominant in parietal regions	A2	5
Alpha frequency (8–12 Hz), dominant in frontal regions	A3	4
Low amplitude, predominantly beta EEG (12–25 Hz) with horizontal SEM	B1	3
Predominantly delta (2–4 Hz) or theta (4–7 Hz) EEG	B2/3	2
K-complexes or sleep spindles	C	1

*VIGALL 2.1* Vigilance Algorithm Leipzig 2.1, *EEG* electroencephalogram, *SEM* slow eye movements

**Table 3:** Scoring criteria of arousal stability score

Scoring criteria	Arousal stability score	
≥ 2/3 of all segments classified as 0 or A1	11	Non-hypoaroused fatigue
≥ 2/3 of all segments classified as 0, A1, A2 or A3	10	
≥ 1/3 of segments from minutes 11–15 classified as B1	9	
≥ 1/3 of segments from minutes 6–10 classified as B1	8	
≥ 1/3 of segments from minutes 1–5 classified as B1	7	
≥ 1/3 of segments from minutes 11–15 classified as B2/3	6	Hypoaroused fatigue
≥ 1/3 of segments from minutes 6–10 classified as B2/3	5	
≥ 1/3 of segments from minutes 1–5 classified as B2/3	4	
≥ 1 C-stage occurred in minutes 11–15	3	
≥ 1 C-stage occurred in minutes 10–6	2	
≥ 1 C-stage occurred in minutes 5–1	1	

### Statistical analyses

Statistical analyses were conducted using SPSS Statistics 23 (IBM corp.; Armonk, NY, USA). Differences between groups were analyzed using nonparametric tests (Chi^2^, Mann–Whitney *U*). The Spearman correlation coefficient was used as measure of association, since tested variables were non-normally distributed. The two-tailed significance level was set to *p* = 0.05. The effect size *ɳ*^2^ was calculated with R version 4.0.2 [63] according to [64]; the bootstrapped 90% confidence intervals are based on 10,000 replications.

## Results

### Descriptive analyses

A total of 102 patients with a current MDD diagnosis according to the ICD-10 and fatigue of clinically significant severity constituted the study sample (see Table 4 for description and scores). Thereof, 57% were unmedicated (i.e., no AD medication for the past 7 days or first dose < 24 h before the recording), 28% were treated with SSRIs and 15% with SSRI combined with other AD medication (see Table 4 for details). No differences between medicated and unmedicated subjects were found concerning the arousal stability score (*M* ± SD: 8.2 ± 1.9 vs 7.6 ± 2.7), BDI-II score (*M* ± SD: 28.7 ± 8.4 vs 30.1 ± 8.8), MFI-20 dimensions, trait (*M* ± SD: 9.2 ± 4.8 vs 8.5 ± 3.3) or state sleepiness (*M* ± SD: 3.5 ± 1.1 vs 3.7 ± 1.3).

**Table 4:** Characteristics of the total sample and the arousal groups; comparison between the hypoaroused fatigue group (arousal stability score ≤ 6) and the non-hypoaroused fatigue group (arousal stability score ≥ 7)

	Total sample (*N* = 102)	Non-hypoaroused fatigue (*n* = 78)	Hypoaroused fatigue (*n* = 24)	Test value	*p* value
Demographic variables					
Age	37.7 ± 12.5 [18–75]	38.4 ± 12.2	35.3 ± 13.5	*Z* = − 1.28	0.201
Gender (F/M ratio)	61/41	45/33	16/8	*X*^2^ = 0.62	0.433
MFI-20 dimensions					
General fatigue	15.0 ± 2.4 [10–20]	14.9 ± 2.4	15.4 ± 2.5	*Z* = − 0.60	0.547
Mental fatigue	14.8 ± 3.2 [4–20]	14.4 ± 3.3	15.9 ± 2.3	***Z = − 2.09***	**0.037**
Physical fatigue	14.0 ± 3.2 [4–20]	13.7 ± 3.3	14.8 ± 2.7	*Z* = − 1.34	0.180
Reduced activity	15.5 ± 2.9 [8–20]	15.3 ± 2.8	16.2 ± 3.1	*Z* = − 1.67	0.095
Reduced motivation	13.5 ± 3.1 [4–20]	13.5 ± 3.1	13.7 ± 3.5	*Z* = − 0.49	0.625
AD medication (yes/no)	44/58	37/41	7/17	*X*^2^ = 2.49	0.114
(Es-) citalopram		25	4		
(Es-) citalopram/mirtazapine		4	1		
(Es-) citalopram/opipramol		1	1		
(Es-) citalopram/trimipramine		2	–		
Venlafaxine		–	1		
Sertraline		5	–		
Arousal stability score	7.8 ± 2.4	8.9 ± 1.6 [7–11]	4.5 ± 1.3 [2–6]		
Arousal related variables					
Time of EEG recording (h:min)	11:02 ± 1:50	10:53 ± 1:45	11:31 ± 2:04	*Z* = − 1.14	0.254
Coffee prior to EEG (yes/no)	61/41	48/30	13/11	*Χ*^2^ = 0.42	0.519
Time of coffee consumption (h:min)	8:05 ± 1:12	8:07 ± 1:15	8:00 ± 1:03	*Z* = − 0.24	0.814
Clinical data					
History of suicide attempts (yes/no)	11/87, *n* = 98	7/67, *n* = 74	4/20, *n* = 24	*Χ*^2^ = 0.94	0.331
ICD-10 diagnosis (F32/F33 ratio)	61/41	44/34	17/7	*Χ*^2^ = 1.58	0.208
Family^a^ history of affective disorders (yes/no)	16/53	11/39	5/14	*Χ*^2^ = 0.14	0.708

Bold values denote statistical significance at the *p* < 0.05 level

Mean ± SD [range]

*MFI-20* Multiple Fatigue Inventory, *AD* antidepressant, *F32* depressive episode, *F33* recurrent depressive disorder

^a^First degree relatives

**Fig. 1:**
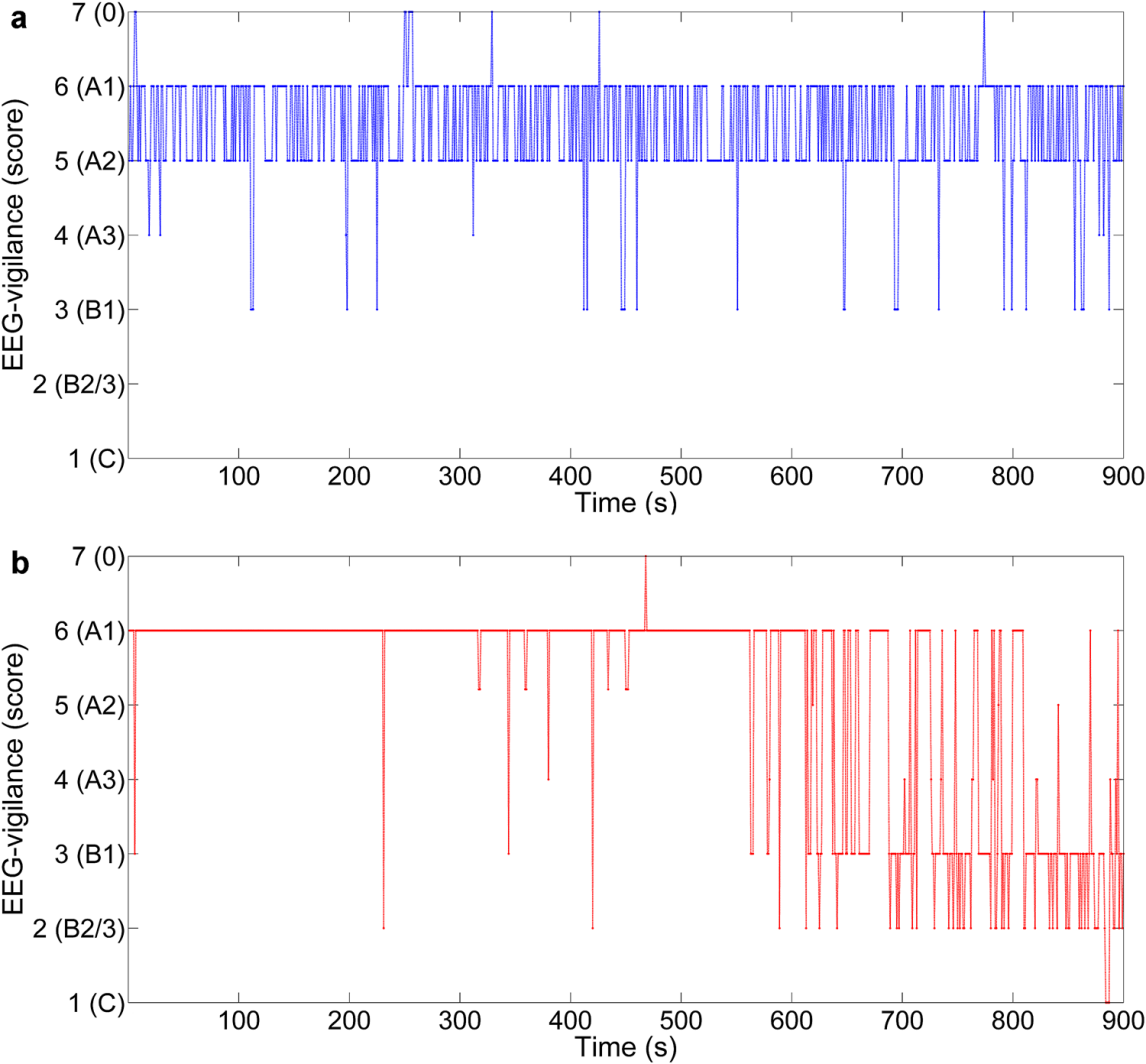
Time course of scored EEG vigilance over 900 consecutive 1-s segments. **a** Subject assigned to the non-hypoaroused fatigue group (arousal stability score = 10) and (**b**) subject assigned to the hypoaroused fatigue group (arousal stability score = 3). Each 1-s EEG segment was classified according to the scoring criteria presented in Table 2

In this sample of fatigued MDD patients, 76.5% stayed in higher arousal states (*n* = 78; non-hypoaroused fatigue group), whereas 23.5% reached arousal states of increased drowsiness or sleep (*n* = 24; hypoaroused fatigue group) during the 15-min EEG recording. No differences concerning arousal biasing variables (see “arousal related variables” in Table 4) were found, as well as no effect of medication on arousal stability score (*M* ± SD; hypoaroused medicated vs hypoaroused unmedicated: 5.1 ± 0.9 vs 4.2 ± 1.4, *Z* = − 1.60, *p* = 0.11; non-hypoaroused medicated vs non-hypoaroused unmedicated: 8.8 ± 1.5 vs 9.0 ± 1.7, *Z* = − 0.50, *p* = 0.62). Representative examples of the time courses of scored EEG vigilance over the entire recording period for either group are presented in Fig. 1.

### Between-group comparisons: BDI-II, ESS, SSS and SF-A/R item comparisons

**Fig. 2:**
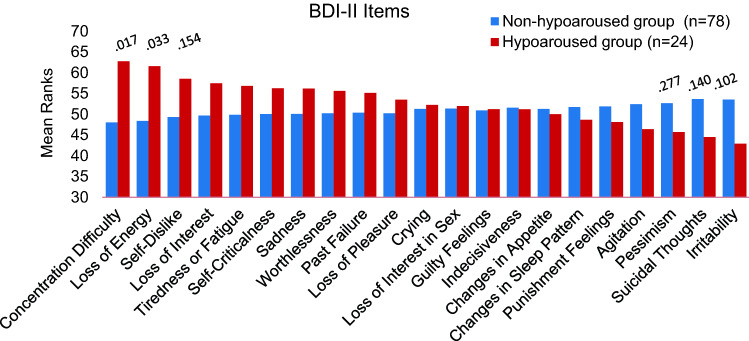
Beck Depression Inventory-II items comparison between the hypoaroused fatigue group and the non-hypoaroused fatigue group. Items are sorted according to mean ranks differences; six smallest *p* values are presented above the bars

BDI-II item comparisons revealed that patients assigned to the hypoaroused group reported significantly higher scores on item “loss of energy” (*Z* = − 2.13, *p* = 0.033; *ɳ*^2^ = 0.044, 90% CI 0.003–0.128) and “concentration difficulty” (*Z* = − 2.40, *p* = 0.017; *ɳ*^2^ = 0.056, 90% CI 0.009–0.139) than patients in the non-hypoaroused group (see Fig. 2).

Although the Mann–Whitney *U* test revealed no significant difference concerning the item “suicidal thoughts” (*Z* = − 1.47, *p* = 0.14), 64% of the non-hypoaroused group vs 42% of the hypoaroused group reported having suicidal ideations (Chi^2^ = 3.81, *p* = 0.051; *ɳ*^2^ = 0.037, 90% CI 0.0008–0.126; “I don’t have any thoughts of killing myself”—0, “I have thoughts of killing myself, but I would not carry them out”—1; “I would like to kill myself”—2; “I would kill myself if I had the chance”—3). Given the clinical relevance of suicidal ideations we further performed an exploratory correlation analysis, wherein the item “suicidal thoughts” and arousal stability score correlated significantly when age, sex and depression severity (total BDI-II score) were included in a partial correlation analysis as control variables (rho = 0.27, *p* = 0.018).

Patients in the hypoaroused group reported significantly higher scores on daytime trait sleepiness and state sleepiness, and experienced significantly higher mental fatigue than patients in the non-hypoaroused group. No group differences were found concerning sleep quality and total time in bed as measured by SF-A/R items (see Table 5).

**Table 5:** Outcome variables for the total sample and comparison between hypoaroused fatigue group (arousal stability score ≤ 6) and non-hypoaroused fatigue group (arousal stability score ≥ 7)

	Total sample (*N* = 102)	Non-hypoaroused fatigue (*n* = 78)	Hypoaroused fatigue (*n* = 24)	Test value	*p* value	Effect size *ɳ*^2^ (90% CI)
Daytime sleepiness						
Trait (ESS)	8.8 ± 4.0 [0–19]	8.3 ± 4	10.4 ± 4	***Z = − 2.20***	**0.028**	0.047 (0.004–0.134)
State (SSS)	3.6 ± 1.2 [1–6] ***n = 99***	3.4 ± 1.2 ***n = 75***	4.1 ± 1.2	***Z = − 2.31***	**0.021**	0.054 (0.004–0.138)
BDI-II score	29.5 ± 8.6 [16–53]	29.2 ± 8.8	30.3 ± 7.9	*Z* = − 0.73	0.468	0.005 (.00–0.05)
SF-A/R						
Sleep quality	2.7 ± 1.0 *n* = 95	2.7 ± 1.03 *n* = 73	2.6 ± 0.7 *n* = 22	*Z* = − 0.23	0.819	0.0006 (.00–0.03)
Total time in bed (h:min)	7:13 ± 1:38 [1:00–11:30] *n* = 100	7:09 ± 1:44 [1:00–11:30] *n* = 76	7:24 ± 1:19 [5:15–10:30] *n* = 24	*Z* = − 0.32	0.749	0.001 (.00–0.037)

Bold values denote statistical significance at the *p*< 0.05 level

Mean ± SD [range]; ESS Epworth Sleepiness cale, *SSS* Stanford Sleepiness Scale, *BDI-II* Beck Depression nventory version 2, *SF-A/R* sleep inventory (refers to sleep during the night before EEG recording)

### Explorative sensitivity analysis

To examine possible effects of the medication on the aforementioned findings, separate analyses for the unmedicated and medicated groups were conducted (see supplementary). Comparably, the hypoaroused group reported higher scores than the non-hypoaroused group on the BDI-II items “concentration difficulty” and “lack of energy” in both medicated and unmedicated patients. However, the difference reached the level of significance only in the unmedicated group. The item “suicidal thoughts” and arousal stability score correlated significantly when age, sex and depression severity (total BDI-II score) were included as control variables in a partial correlation analysis in the unmedicated (rho = 0.29, *p* = 0.03) but not in the medicated (rho = 0.06, *p* = 0.69) group. With or without medication, the hypoaroused group reported higher state and trait sleepiness, and higher mental fatigue, although only the difference in state sleepiness within the unmedicated group reached statistical significance.

## Discussion

In the present study, we aimed to explore whether stratifying MDD patients with fatigue by brain arousal, as assessed with an EEG-based resting-state measure, would reveal two subgroups with distinct clinical features. We found that the subgroup with downregulated brain arousal (hypoaroused group) scored significantly higher on BDI-II items “concentration difficulty” and “lack of energy”, on state sleepiness (accessed by SSS) and trait daytime sleepiness (accessed by ESS), and on the MFI-20 dimension “mental fatigue”. In contrast, suicidal thoughts, as measured by one of the BDI-II items, were more frequent in the non-hypoaroused subgroup.

Regarding the BDI-II measure of depressive symptoms, significantly more pronounced concentration difficulties in the hypoaroused group may have resulted from the instability of brain arousal which interferes with the ability to concentrate, and hence contributes to greater mental fatigue, as measured by the MFI-20. Notably, “concentration difficulties” was the most predictive for antidepressant response among 31 clinical and demographic features, as reported recently in a study using a machine learning approach [54]. Moreover, patients in the hypoaroused group complained about a greater loss of energy, despite similar levels of physical and general fatigue. This may reflect the fact that within typical (hyperaroused) depression, patients do not suffer from lack of energy or drive but from inhibition of drive with exhaustion and high inner tension—a completely different psychopathological syndrome [5, 6].

The between-group comparison of the BDI-II item “suicidal thoughts” was marginally significant (*p* = 0.051), with suicidal ideations more often reported by the non-hypoaroused group. Subsequent exploratory correlation analyses revealed a positive correlation (rho = 0.27, *p* = 0.018) of brain arousal with the propensity towards suicidal ideation after adjusting for age, sex, and severity of depressive symptoms, which are known parameters associated with suicidal tendencies [55]. This finding supports the view of hyperarousal as a potential pathophysiological mechanism associated with suicidal ideation [56], and is in line with a recent quantitative EEG sleep study by Dolsen et al. [57]. The authors provided evidence, after adjusting for age, sex, depressive and insomnia symptoms, that hyperarousal during sleep and suicidal ideations may be associated in MDD patients. Therefore, our results support the view of an upregulated brain arousal as a worthwhile target in clinical research which may help to better understand the mechanisms underlying suicidal ideation [58].

Regarding subjective trait daytime sleepiness and state sleepiness at the time of the EEG, increased sleepiness was associated with downregulated arousal, as it has previously been reported for healthy individuals [28]. Both findings support the validity of the VIGALL classification. It is conceivable that patients with downregulated brain arousal were drowsy or fell asleep due to poor sleep quality the night before the recording, since nighttime sleep can affect daytime vigilance, however, no between-group differences of sleep parameters were found. This indicates that group differences concerning arousal and state/trait sleepiness are independent of the sleep characteristics in the night preceding the EEG recording.

Despite the profound fatigue all patients experienced, less than a quarter of the sample (23.5%) reached arousal stages of increased drowsiness or fell asleep within the 15-min eyes-closed EEG during quiet rest. This proportion is markedly low compared to those found in (1) a comparable healthy sample without fatigue (48.3%; [37]) and (2) a 10-years-older sample with cancer-related fatigue of comparable severity (59%; [43]). The fact that only a minority of fatigued patients in our sample shows a pronounced decline of brain arousal is in line with earlier studies reporting an upregulated brain arousal in patients with MDD as assessed with VIGALL [37, 41, 42].

There was no effect of medication on arousal neither within the total sample, nor within the arousal groups. The effect of hypoarousal in medicated and unmedicated patients showed the same direction as found in the total sample; nonetheless, the level of significance was only reached within the unmedicated group.

We suggest stratifying patients along the dimension of brain arousal. Differentiating fatigue into a hypo- and hyperaroused subtype in depressed patients may be of relevance for personalized treatment. There is some evidence that treatment with psychostimulants may be beneficial in patients with hypo- but not hyperaroused fatigue (reviewed in [6, 45]). Furthermore, a better response to antidepressants such as selective serotonin reuptake inhibitors has been observed in MDD patients with upregulated brain arousal [46].

Several limitations of the current study should be mentioned: first, the assessment of sleep behavior of the night preceding the EEG recording was based on subjective ratings. Second, the MFI questionnaire that had not yet been standardized for the depressed populations required a cut-off definition for scores representing fatigue of clinically significant severity. Previous studies support our choice of the cut-off [21, 59], e.g., [60] reported that 93.8% of their depressed sample (cf. 93.6% in our sample) complained about clinically significant fatigue as assessed with the Fatigue Questionnaire [61]. Third, inclusion of medicated patients can be seen as a limitation, since fatigue has been described as an adverse reaction to antidepressant treatment (for review [62]). However, in our sample, no differences concerning fatigue scores were found between medicated and unmedicated patients.

Stratifying fatigued MDD patients by arousal may lead to subgroups, which are more homogeneous concerning pathophysiology and symptomatology. Brain arousal may be a worthwhile target in clinical researchofor better understanding the mechanisms underlying suicidal tendencies and to improve treatment response.

## Electronic supplementary material

Below is the link to the electronic supplementary material.

Supplementary file1 (PDF 183 KB)
