# Supplementary material for: Fatigue and brain arousal in patients with major depressive disorder
Source: Eur Arch Psychiatry Clin Neurosci. 2020 Dec 4;271(3):527–36. doi: 10.1007/s00406-020-01216-w (PMC7981331; doi:10.1007/s00406-020-01216-w)
Supplement: Supplementary file 1 — Supplementary file1 (PDF 183 KB) [file 406_2020_1216_MOESM1_ESM.pdf]

Supplementary Mann–Whitney *U* test for group differences (hypoaroused vs non-hypoaroused) in medicated and unmedicated patients

	Unmedicated (n=58)					Medicated (n=44)			
	Hypoaroused fatigue (n=17)	Non-hypoaroused fatigue (n=41)	Test value	*p* value	Effect size η^2 (90% CI)	Hypoaroused fatigue (n=7)	Non-hypoaroused fatigue (n=37)	Test value	*p* value
BDI-II									
"concentration difficulty"	2.00±0.61	1.56±0.74	Z= –2.07	**0.038**	0.074 (0.006-0.204)	2.00±0.58	1.65±0.68	Z= –1.26	0.207
"lack of energy"	1.88±0.60	1.49±0.71	Z= –2.04	**0.042**	0.072 (0.005-0.204)	1.86±0.69	1.62±0.55	Z= –0.88	0.377
Daytime sleepiness									
Trait (ESS)	9.7±2.6	8.0±3.5	Z= –1.75	0.081	0.053 (0.001-0.176)	12.0±6.2	8.7±4.5	Z= –1.63	0.104
State (SSS)	4.2±1.2	3.4±1.3 n=38	Z= –2.36	**0.018**	0.101 (0.008-0.228)	3.7±1.3	3.5±1.1	Z= –0.36	0.722
MFI-20									
Mental fatigue	15.9±2.7	14.7±3.2	Z= –1.21	0.225	0.025 (.00-0.140)	16.0±1.3	14.1±3.5	Z= –1.63	0.103

Mean±SD; bold values denote statistical significance at the *p*<0.05 level

BDI-II Beck Depression Inventory version 2, *ESS* Epworth Sleepiness Scale, *SSS* Stanford Sleepiness Scale, *MFI-20* Multiple Fatigue Inventory
